# “You Feel Like You Kind of Walk Between the Two Worlds”: A Participatory Study Exploring How Technology Can Support Emotion Regulation for Autistic People

**DOI:** 10.1007/s10803-021-05392-z

**Published:** 2022-01-11

**Authors:** Lauren Gillies-Walker, Naeem Ramzan, Jean Rankin, Emy Nimbley, Karri Gillespie-Smith

**Affiliations:** 1 The University of The West of Scotland, Paisley High Street, Paisley, PA1 2BE UK; 2 The University of Edinburgh, Edinburgh, EH8 9AG UK; 3 Department of Clinical and Health Psychology, School of Health in Social Science, The University of Edinburgh, Old Medical School, Doorway 6, Edinburgh, EH8 9AG UK

**Keywords:** Autism, Technological supports, Communication, Emotion regulation, Anxiety

## Abstract

An increasing amount of technological solutions aiming to support emotion regulation are being developed for Autistic people. However, there remains a lack of understanding of user needs, and design factors which has led to poor usability and varied success. Furthermore, studies assessing the feasibility of emotion regulation technology via physiological signals for autistic people are increasingly showing promise, yet to date there has been no exploration of views from the autistic community on the benefits/challenges such technology may present in practice. Focus groups with autistic people and their allies were conducted to gain insight into experiences and expectations of technological supports aimed at supporting emotion regulation. Reflexive thematic analysis generated three themes: (1) communication challenges (2) views on emotion regulation technology (3) ‘how’ technology is implemented. Results provide meaningful insight into the socio-emotional communication challenges faced by autistic people, and explore the expectations of technology aimed at supporting emotion regulation.

## Introduction

Autism is defined as a complex neurodevelopmental condition characterised by social interaction and social communication difficulties, as well as repetitive behaviours and focused interests (American Psychiatric Association, 2013). In recent years, the neurodiversity movement has brought about a huge shift in Autism research, theory, and practice; viewing Autism as part of a neurodiversity continuum rather than a disorder (Leadbitter et al, 2021). Although both Autistic and non-Autistic people need and want meaningful friendships (Cresswell et al., 2019), different styles of communication often result in bi-directional issues in understanding and empathising with each other (Crompton et al., 2020; Sedgewick et al., 2019). These different ways of communicating have been referred to as the double empathy problem (Milton, 2012). Autistic socio-emotional communication is unique to each individual and dependant on multiple factors including level of ability and stage of development (Lodge et al., 2019).

High rates of anxiety in Autism have been linked to emotion dysregulation (for meta-analysis see, Van Steensel & Heeman, 2017) and the advancement of new and innovative emotion regulation technologies have resulted in great potential for use within support strategies (for reviews see: Jaliaawala, & Khan, 2020; Lee et al., 2018; Boucenna et al., 2014). Real time emotion regulation technology via physiological signals has also been proposed as a feasible method of self emotion-regulation (Williams & Gilbert, 2020). However, to our knowledge no studies have assessed views from the Autistic community regarding the benefits or challenges these devices may present in practice.

### Emotion Regulation in Autism

Emotion Regulation refers to the process of effectively managing and modifying the expression or experience of emotions (Thompson, 1994). Difficulties in emotion regulation is a common issue for many Autistic people and has been found to result in increased anxiety, depression, anger and physical health outcomes (Cai et al, 2018; Mazefsky et al., 2014; White et al., 2014; Zantinge, et al., 2017). Conner et al (2020) found that emotion regulation difficulties in Autistic people significantly predicted elevated levels of anxiety and was associated with suicidal ideation. Around 50% of Autistic individuals experience higher levels of social anxiety (Benevides et al., 2020a, b; Maddox & White, 2015) and sensory overload (Rodgers & Ofield, 2018) than non-autistic people. Those with more complex needs e.g. intellectual disability, limited verbal ability and sensory overload, are particularly at risk (Samson et al., 2014). High anxiety and frustration are often evidenced by behaviours frequently termed as ‘challenging behaviours’ such as aggression, self-harm, harming others or running away (White et al., 2009; O’Nions et al., 2018).

Indeed, autistic people also tend to experience more social isolation and depression than non-autistic peers (Lai et al., 2017) and have reported many obstacles when trying to access support for mental health (Crane et al., 2019) which can lead to poorer quality of life (DaWalt et al., 2019). This indicates an urgent need for support strategies which not only cater to the wide variety of abilities and needs of Autistic people, but ultimately improve mental health for both individuals and their care givers (McGuire et al., 2016; Suzuki et al., 2019).

### The Use of Technology to Support Emotion Regulation

Many studies have identified advantages of technology for Autistic people and how they can improve communication in daily life (Zhang et al., 2020). For instance, interactive technologies can be more predictable than humans, as direct social interaction is not required and they can give feedback or consistent rewards (Grynszpan et al., 2014; Rapp et al., 2018). Jeffs et al., (2005) found that parents and children can learn from each other when engaging with and interacting using assistive technology. Common communication aids include picture communication systems via applications (apps), speech generation aids, games via mobile phones, tablets or virtual reality devices that mimic social scenarios, allowing users to ‘practice’ social interactions. In a systematic review, Pérez-Fuster (2017) found that handheld devices such as tablets and smartphones were the most popular technologies for enhancing daily living skills for Autistic people. Although Autistic individuals tend to use technology for entertainment more than non-autistic peers (Mazurek & Wenstrup, 2013), less is known about how Autistic people use technology for social and emotional communication and management, e.g. regulating emotion and reducing anxiety. Systematic reviews (see Benevides et al., 2020a, b; Berggren et al., 2017) suggest that future research should aim to better understand the lived experience of technology to support socio-emotional communication in Autism.

Several studies have explored the feasibility of non-invasive wearable technologies for emotion regulation strategies via physiological signals (Puli & Kushki, 2019; Benssassi et al, 2018; Taj-Eldin et al., 2018; Koo et al., 2018). Results indicate that increasing awareness of emotional states via technology, may allow the individual or a caregiver to take actions that would reduce stress or frustration, ultimately having the potential to improve emotion regulation, reduce anxiety and prevent ‘challenging behaviours’. However, in a survey of wearable technologies for Autism interventions, Williams and Gilbert (2020) found that 90% of identified studies viewed Autistic traits as deficits to be ‘fixed’ and only 10% of technologies focused on supporting sensory regulation, emotion regulation, executive function or communication. This suggests that many wearable technology studies have been based on outdated theoretical models of Autism (i.e., a disorder to be treated).

Therefore, despite showing promise (Taj-Eldin et al., 2018; Odom et al., 2015), it is not surprising that the success of emotion regulation technology has been varied and usability/uptake remains poor. This may also be a result of a lack of participatory methods that consider user satisfaction and how design features can address individual needs. Fletcher-Watson et al., (2016) shed light on such inconsistencies in the reported success of technology and suggest that little work has directly connected technology design with user experience or outcomes (referred to as participatory design). Furthermore, the lack of methodologically robust evaluation methods to assess technologies may also explain the varied results; with only a minority of studies involving control groups or follow-up evaluations (Benssassi et al., 2018; Grynszpan et al., 2014). Addressing this problem places high demands on technological supports as differing levels of ability and varying rates of development must be taken into account at the design stage. Thus, a participatory exploration of views from the Autistic community regarding experiences and expectations of emotion regulation technology is essential to provide a basis for designing future ethical and inclusive technological supports.

*This Paper Aims to:*

- Provide meaningful insight into experiences and expectations of technology that aims to support emotion regulation
- Explore participant’s views on the benefits and challenges of real time emotion regulation technology via physiological signals
- Provide a basis for improving individualised and interactive technology aiming to support emotion regulation for Autistic people

## Methods

### Design

In line with the aim of understanding complex phenomena and improving quality of life (Smith, 2003), a qualitative participatory design was chosen and 8 semi-structured focus groups were carried out. Semi-structured questions provided participants with more control over the discussions and how they voiced their experiences (Stewart & Shamdasani, 2014). These lasted around 1hr with interviews digitally recorded for transcription, before reflexive thematic analysis was conducted on anonymized transcripts by the researcher. Most participants were not aware of questions before focus groups were carried out, however two Autistic participants requested to have some examples beforehand.

### Participants

Through opportunity sampling, thirty-four participants with a wide variety of knowledge and experience of Autism were recruited. Participants included; Autistic adults, parents of Autistic children and adults, health/social care staff who support Autistic adults in a community care setting, and educational staff who support Autistic individuals at college level. The selected groups ensured multiple perspectives were obtained that reflect both the heterogeneity of Autism and technology use across a wide variety of contexts (home, education, and community care). Furthermore, health/social care staff, parents of Autistic children and educational staff had experience supporting autistic individuals across the spectrum, ranging from moderate to high IQ’s, to those with highly complex needs (e.g. various levels of adaptive functioning, lower IQ, more difficulties in emotion regulation, ‘challenging behaviours’). This therefore ensured insight into Autistic experiences reflecting a broad range of IQ. Ultimately, the diverse range of experiences of participants allowed insight into technology use across a wide variety of needs and abilities.

### Procedure

Written and verbal consent was obtained from all participants before each focus group. Due to COVID19 two focus groups with Autistic participants were carried out online. Two participants (one who was non-speaking and another with sensory needs) chose to take part via the zoom chat box. This may have influenced amount of information provided, however online focus groups were also noted as being a preferred method by the Autistic participants' and inclusive adaptions such as this enable Autistic individuals to take part in research who otherwise may not. All participants were given contact details to review the results.

### Focus Group Questions

Questions in each focus group were split into two domains: (1) any type of technology that aims to support social and emotional communication. (2) Emotion regulation technology. This was to ensure participants had a chance to share more general experiences and expectations, before narrowing the discussions specifically to emotion regulation technology. Participants discussed both their own experiences and voiced opinions about emotion regulation technology for the wider Autistic community. The researcher presented some examples of how emotional regulation technology may be used in practice (e.g. real time technology that detects physiological signals and is able to prompt users to self-regulate emotions, ultimately with the potential to reduce anxiety/stress).

Table 1 below shows the Autistic group demographics and Table 2 shows characteristics (gender and age range) for all participants.

**Table 1:** Autistic group participant demographics

Participant	Age	Gender	Ethnicity	Occupation
1	23	Female	White/Caucasian	Student
2	24	Male	White/Scottish	Student
3	42	Male	White/Scottish	Disability Campaigner
4	31	Male	White/Scottish	Disability Campaigner
5	35	Male	White/Scottish	Student
6	38	Female	White/British	Student
7	23	Female	White	Student
8	21	Male	White/Scottish	Student

**Table 2:** All participant characteristics (n = 34)

Focus group sample	Total N (number of woman)	Age range
Autistic Adults	9 (4)	21–42
Parents of Autistic Children and Adults	9 (8)	30–52
Health and Social Care Staff (supporting Autistic individuals in a community care setting)	14 (10)	25–60
Educational Inclusive Learning Staff (supporting Autistic individuals at college level)	2 (2)	25–40

### Qualitative Analysis

Each focus group was facilitated by the primary researcher and was digitally recorded before being listened to and transcribed. Analysis took a data-driven approach using reflexive thematic analysis, facilitated by a systematic six phase process outlined by Braun et al., (2019). This methodology was chosen as it enables an ‘open and organic’ process rather than basing themes on theory or previous research (Braun & Clarke, 2006, 2020). Although the analytical process was facilitated by the 6 phase process outlined below, no coding guidebook was used. Instead, themes were developed according to patterns of shared meaning. Table 3 below outlines the analytical process.

**Table 3:** Reflexive thematic analysis (Braun et al., 2019)

Phase	Analysis strategy
1	Each focus group was read several times for familiarisation and initial ideas were noted. All focus groups were transcribed
2	Interesting features of the data were highlighted across the entire data set to produce initial codes and a table of ‘key quotes’ was produced for each focus group
3	The NVivo data management program was used to input data, and an ‘open and organic’ (Braun & Clarke, 2020) approach to coding the data was adopted. This involved categorising all focus group transcripts into “nodes” (the thematic feature in NVivo10) then generating themes according to patterns of shared meaning
4	Themes were reviewed and refined. A thematic map of the data was produced
5	Themes were defined before being checked by another member of the research team to confirm consistency
6	Final write up of the analysis and report. Data extracts were chosen to provide evidence and highlight the prevalence of each theme

## Results and Discussion

Overall, three themes were identified within the data (1) communication challenges (2) benefits/challenges of emotion regulation technology (3) ‘*how*’ technology is implemented. The following section will discuss the shared meaning behind each theme and sub theme. In order to protect the identities of participants, pseudonyms are used throughout (Table 4).

**Table 4:** Main themes and sub-themes

Theme	Sub-themes	Examples of codes
1. Communication challenges	Emotion processing challenges	Experiences of ‘challenging behaviours’, experiences of technology, experiences struggling with emotion regulation, camouflaging, socializing, positive experiences, negative experiences
	Person-centered Technologies are needed	Individualised technology, co-participantion, different needs and abilities, adaptable designs
2. Benefits & challenges of emotion regulation technology	Benefits of Emotion Regulation Technology	Positive design features, positive experiences, self emotion-regulation
	Challenges of Emotion Regulation Technology	Funding issues, usability, lack of knowledge about what technology is available, ethical issues for future developments,
3. *‘How’* technology is implemented	Training is needed	Experiences of lack of training, poor training, how training could be improved
	Defining individual goals and needs	Positive example of support strategies, implementation, life goals/individual needs wants/desires, promoting independence, meeting individual needs, consistent staff, co-participatory methods, focus on environment/context in implementation strategies for those with more complex needs

### Theme 1: Communication Challenges

A wide range of communication difficulties typically associated with Autism and the impact on social interactions and emotional well-being was discussed extensively across all groups.

#### Sub-theme: Emotion Processing Challenges

Social and emotional challenges can vary widely for Autistic people, depending on individual ability and stage of development. Difficulties expressing and regulating emotions were discussed in detail. Parents expressed how this can impact mental health and well-being of families and carers:

Sarah: She looks perfectly fine and she’s behaving perfectly fine..except…she’s not

Wendy: Do you feel like you’re walking on egg shells in your own home?

Sarah: Oh Yeah..yeah, we’re at a point with Lucy where…like..she’d just come home from school n she’s contained it n then she’ll come home n she’ll just go..off her nut

Sarah went on to explain how her daughter may seem okay outwardly, however struggles emotionally internally:

Sarah: Now we’re at a point where..we can’t..she hides it so well, the anxiety and everything that – we haven’t got a clue! If she’s feeling a certain way or she’s at a certain point, we don’t see it. Her facial expressions, her mannerisms, everything. Sometimes the prodding can lead to a big explosion

Sarah’s experience with her daughter is not uncommon as research has shown that an autistic person can appear outwardly calm to those around him or her, while having an unusually high resting heart rate – a common indicator of high physiological arousal (Kaliouby et al., 2006; McDonnell et al., 2015). Sarah’s experience reflects what researchers have identified as ‘camouflaging’, causing internal states (often in turmoil) to be masked. Several researchers have documented that camouflaging is more common in women (Hull et al., 2020; Lai et al., 2019) and that the stress of maintaining a ‘public mask’ can lead to more problems at home (Anderson et al., 2020). Due to emotions being hidden so well in public, emotional communication challenges may go unnoticed or without further exploration and have been linked to depression both in Autistic men and woman (Cage et al, 2018; Lai et al., 2017).

One Autistic participant shared how he currently deals with emotional challenges:

Harry: So I’ll try and shut myself away…so that when I do go into, if it’s a fit of rage…I wont endanger anyone

These experiences further stress the need for useful and effective strategies to support Autistic people in self-regulating emotions.

Educational staff shared experiences of Autistic students struggling to socialize with peers and interacting in a classroom environment. They also felt that students can be reluctant to try new technology due to lack of training or preferring to have the support of staff members.

Alice: I would say the issues have been more about actually..em interaction with other peers in their class n things.

Jill: A lot of the emotional support I gave last year was about social interaction

Additionally, some Autistic participants commented on the lack of understanding from non-autistic people in social/emotional interaction:

Connor: I was just going to say like I sometimes feel like the same kind of way, where I feel like I’m not really the one who’s Autistic, it’s kind of other people and their lack of understanding.

Such challenges in social interactions between autistic students and their peers may be explained by ‘The double empathy theory’ proposed by Milton. This suggests that the social and emotional challenges many autistic people report (i.e. friendship and socializing) are not solely due to autistic cognition, but a breakdown in mutuality and understanding that can happen between people who communicate in different ways. Crompton et al., (2020) found that Autistic people shared information in social interaction with other Autistic people just as well as non-autistic people do with other non-autistic people, suggesting that *differences* in communication impact relationships between autistic and non-autistic people. This new approach to understanding the different communicative styles between autistic and non-autistic people is crucial for developing and designing future support strategies using technology.

Carers shared their experiences of how emotional challenges can impact behavior and how they felt technology could be integrated into support strategies to benefit people:

Ann: he can’t tell us what’s going through his head..you can’t tell how he’s feeling. He could look really happy but he could be ready to have a meltdown

Jean: Yeah one gentlemen I support is very quick. And we always know there’s something causing it but he goes from one extreme to another very quickly.

Ann: You want to get in before the behaviour starts..before it escalates..we could go in before to offer reassurance..a distraction..or for other people its withdrawal..give them their own space

Alison: But then Harry’s always got a trigger to make him feel these ways

Gary: Yup..and that might help us determine what the trigger is before..…we know there might be a pattern but we just can’t see it

Carers discussed how technology that aims to support individuals in expressing their emotions or record patterns in the behaviours would be more beneficial than the current method of recording their emotions on paper. In line with Laurent et al., (2018), this highlights how emotion regulation technology may help parents or carers establish patterns in behaviors, key points of high anxiety and potential anxiety triggers which would be invaluable in supporting the autistic person e.g. giving them space, in altering the sensory environment, or to avoid situations they may find particularly stressful.

#### Sub-theme: Person-Centered Technologies are Needed

One of the key aims of the study was to provide meaningful insight into experiences and expectations of technological supports that aim to regulate emotions. Participants across all groups expressed the need for person-centered and adaptable technology to cater to a wide variety of abilities and needs.

Autistic participants highlighted how technology is often more geared towards individuals with an intellectual disability or complex needs and rarely aimed at those with a higher IQ:

Harry: It’s as if they’ve put people with all Autism under the one IQ

Harry: All the Autism apps are geared to the one specification

Andrew: Yeah there was certainly an issue with products and where they were putting where it was pitched in the spectrum, if that sense?

Alex: You feel like you kind of walk between the two worlds almost…like you’re not quite servere, so you’re not at that point on the spectrum where you need a lot of support that you’d get if you were

Kirsten: And yeah lower support needs are often ignored (not like people with high support needs, we just kinda get left behind)

Katie: nice sensory technology is a big thing for me

Indeed, IQ level will impact what technology can be used and how it is used, which further highlights the importance of adaptive designs. Participants discussed potential solutions and suggested that being able to input design preferences into technology prior to use would be useful (e.g. adaptive lighting, sounds, fonts, colours on interfaces). These experiences reflect the heterogeneity of Autism and evidently, must be addressed in the design, development, and implementation of technologies.

It was also agreed across all groups that in order for technology to be successful, co-participation at every stage of the research process is vital:

Alex: If your gonna make something for someone ask them what they want don’t just spit out something and go here’s what I made. And it’s everywhere whether its an app or technology or even the way events are organised or how people write up things. Like the amount of papers where people claimed to have made something for learning disabilities or you know…and its like have you ever had it tested have you ever used it with anyone??

Autistic participants also agreed that technological designs should promote independence, rather than try to ‘mask’ or ‘fix’ Autism.

Katie: This can be a hard thing with technology, the more it helps and masks my autism, the less people will understand that I still struggle and the amount of difficulty I face navigating and communicating in a neurotypical world

### Theme 2: Views on Emotion Regulation Technology

Since studies assessing the feasibility of emotion regulation technology for Autistic people are increasingly showing promise, yet with varied success in practice; a key aim was to explore participant’s views on this (specifically the benefits and challenges). Participants were asked about their experiences with any type of technology that has helped with regulating emotions and their views on real time emotion regulation technology based on physiological signals.

#### Sub-theme: Benefits of Emotion Regulation Technology

Participants considered many positive aspects of technology use:

Tom: Even if it gets broken, there’s a procedure to fix it. It doesn’t wake up in a bad mood..it doesn’t have a bad nights sleep….people are inconsistent (as far as you try and be consistent).

This highlights how Autistic people enjoy and interact well with technology as it is consistent and reliable. Parents also evaluated the use of iPad and YouTube videos to regulate emotions:

Alison: but he’ll repeat the same segment, the same 30 seconds to a minute over and over again n it like ok I know what’s gonna happen…he’ll talk along with it. So he’ll get language that way so he uses it to help relax and calm….regulate himself … so yeah, it’s a calming, it’s a comfort for him

Here, Alison highlights how technology can be used to engage in repetitive behavior (stimming) which is often a coping mechanism or an anxiety reduction technique instinctually employed by autistic individuals (Kapp et al., 2019). Since research on technology as a stimming aid is limited, further exploration on appropriate and effective technological supports aiming to regulate emotions and reduce anxiety is needed (Kapp et al, 2019).

Participants were asked about their views on emotion regulation technology based on physiological signals and gave examples of how such technology might be useful:

Milly: Obviously it doesn’t change anything, it doesn’t fix anything, maybe if you’re having a stim moment or if you’re feeling anxious but I suppose it does let you know that you need to take some time out.

Norma: I’d like something similar to what you said..emm.. something that he can self regulate, tell people how hes feeling, something that’s an app that somehow connects with a colour, so he can pick a picture that says how he’s feeling and people know without it being a big song and dance and so he’s not singled out…something that connects to the phone…from the internal to the external

Connor: I don’t think technology or anything will get to the point where it will be the solution to all our problems or whatever, but I feel like you know, having the tools at my disposal and to use them however I can to make my life easier

One participant also shared that he will use his heart rate monitor on his watch to regulate emotional well-being:

Connor: It’s funny you say that because I’m currently wearing my watch, which detects my heart rate and I do periodically look at it to actually see where I am to actually try to establish whether or not I am ok

#### Sub-theme: Challenges of Emotion Regulation Technology

There was consensus among participants regarding difficulties in getting access to not just emotion regulation technology but technology in general (either through lack of funding or knowledge). Many Autistic people supported in their own homes within a community care service and who have more complex needs (e.g. non-speaking or severe learning difficulties) use technology such as door alarms, bed alarms or wall sensors. However, care staff highlighted that access to any other technology specifically focused on emotion regulation is extremely limited due to personal budgets or funding:

Ann: *nods* yi can try but we don’t have anything

Jean: We don’t have anything signed off to say its accessible

Ruth: It’s a bit of a fight to get resources

This may explain differences across groups, e.g. why parents using technology (such as iPad, tablets, and mobiles) at home with their children expressed more positive experiences than care staff within community settings. This highlights the impact of socioeconomic factors (e.g. parent education, employment status or income) when accessing technologies. The data therefore highlight the need for low cost technological supports if they are to be accessible to a wider population.

Knowledge of different types of emotion regulation technology available was also highlighted as a barrier:

Gary: I think the barriers that tend to come up is that some of the people we support don’t have access to that kind of technology and if they do it’s not necessarily the people we support who use it, it’s the staff members who use it on behalf of them to do certain things.

Milly: I don't think I'm aware of any but if I were I don't know how and when I would be able to use it

Jean: N its limiting the guys we support as well cause..they don’t get taught a lot about different aids and adaptions n how they can use some technology

Here, it is clear that access and knowledge about emotion regulation technology can greatly impact expectations and use. Moving forward it is essential to recognize this diversity and how socio-economic factors (such as income) can influence technology use/perceived ability to use technology, especially since Autistic people are less likely to have well-paying jobs compared to non-autistic people (Pellicano et al, 2013).

Educational staff reported experiences with students who would rather have human–human interaction for emotional support.

Alice: I don’t think it can replace human interaction

This may also be indicative of technology that is to date implemented without being co-produced by the autism community who would be able to advise on systems that were more intuitive for autistic groups. In addition to socio-economic factors there were also concerns about media use due to the scare campaigns about the effects of screen time and lack of clear guidance on technology and media. Parents of children who already struggle with social interaction may be more anxious about their child using technology for long periods (Valkenburg & Peter, 2009) since media often communicates the disadvantages of long screen time.

Sharon: I feel like it’s a need in our house as well, if they don’t have restrictions, if I don’t have any restrictions around using the technology, then it could become a 6 or 8 hour day on a device…

When asked about their views on real time emotion regulation technology based on physiological signals, positive aspects were highlighted, however some participants expressed important ethical concerns which must be prioritized and addressed in order for such technology to be beneficial to Autistic people:

Emma: Also, it freaks me out a bit that a device would be monitoring and then an intervention based on that..the ideal states seems veering into territory of a state that is optimal Vs state that is suboptimal - again, something that has been weaponised against neurodiverse people

Emma: I think the ethics issue is important but the only way to do that is creating with actually autistic people

These views place emphasis on the need for emotion regulation technology that does not intend to replace Autistic traits with ‘ideal states’. Instead, focus must be placed on developing technologies that give full autonomy in order to improve emotion regulation in a way that is useful and effective based on unique abilities and needs.

A summary of differences in opinions of real time emotion regulation technology across all groups are outlined below (Table 5).

**Table 5:** Differences in views of real time emotion regulation technology via physiological signals

Focus group	Summary of views	Evidence/quotes
Autistic individuals	Highlighted ethical challenges around detection of emotional state e.g. consent. Some individuals might not want their internal state to be detected by others. Felt that such technology might be more useful for individuals with more complex needs but not necessarily for those with higher IQ. Highlighted lack of focus on technological solutions for Autistic people with a higher IQ Some participants said they monitor heart rate on a smart watch as a form of emotion regulation	Harry: I found that it’s geared more towards those who havn’t got a high IQ level Kirsten: I would be interested to see if it worked. I do sometimes look for signs I'm in the 'rumble zone' for a meltdown so that I can get into a safe space (usually things are sensory but sometimes emotional dysregulation). Connor: okay, well emotion regulation definitely, the heart rate thing really sounds good to me, that’s right up my alley, because again my biggest issue has always been, oh my gosh I’m really panicky you know but I don’t realise it until I’m 30 min in. So that does actually sound like something that would be really useful for me Emma: so for me, the aspect of tracking mood has been quite useful- something that links in with my diary and also mood tracker could be good because knowing patterns for times of days or things that trigger moods/meltdowns could be helpful Connor: Exactly yeah, and it kind of takes the pressure off you to identify that yourself. You know, ehm, which is one less thing to worry about. So I definitely think there would be place for it
Parents	Generally positive about the use of technology to help their child regulate emotions. Suggested that real time technology would be useful to provide insight into physiological state in order to identify patterns in behaviour	Sharon: It would be really good to get patterns..for me to help learn his behaviour..yeah, that sort of thing would be helpful!…but that’s purely just in my situation
Support workers	Positive about the use of real time emotion regulation technology to reduce ‘challenging behaviours’. Liked idea of being able to intervene early e.g. alter sensory environment. Also placed emphasis on individuals using technology to self-regulate emotions/improve independence Highlighted a potential disadvantage: although the individual or care-giver would be made aware of the internal state, they may not know the trigger and still struggle to respond appropriately, indicating that there will still be some guesswork involved in reducing anxiety/frustration	Gary: So if there was something that could indicate that then we could go in really quickly with distraction or give him his space sooner..cause just now we don’t always get it right, we don’t always pick the right time to intervene Gary: We’ve got Micheal for example; he could maybe see something and get quite distressed so that way we’d be able to pick up on patterns or triggers
Educational inclusive learning staff	Sceptical about real time technology replacing human emotional contact in an educational context. Felt that Autistic students prefer human–human contact due to barriers to technology use e.g. lack of training/poor design features	Ann: How can an iPad replace me listening to someone everyday

### Theme 3: ‘How’ Technology is Implemented

Participants agreed that placing more of a focus on *how* technology is implemented in the home, care settings or in education may be key in developing technological supports that are more in line with individual needs and ultimately more useful. The steps taken (e.g. training, follow-up evaluation meetings, consistency within staff teams) to ensure technology is implemented appropriately and effectively according to individual needs/life goals is often overlooked both within academic research and practice. Participants discussed ways in which they felt the overall success of technology in supporting emotion regulation could be improved:

#### Sub-theme: Training is Needed

Participants across all groups expressed how there is little or no training on how to use technology in general:

John: They can’t just really give you a device n say right here you go crack on now…it’s down to money and making the product and its down to education and training everybody to use it then the research behind using the product aswell

Katie: The training was always hard because it is with a new person you do not know with a new thing and I just struggle to process a lot of it, so I feel a lot of it is about learning on your own

Jill: students need to be given official training….the training that we receive..I don’t think is official enough, either

Alice: I have to admit, my experience would be, most of the students that I work with..if you try n introduce assistive technology they’re not..their quite reluctant.

Alice went on to explain how staff are only given ‘a shot’ at using technology if they have a question and only in their ‘down time’. Lack of training for both staff and students may explain why students are ‘quite reluctant’ to use it. Thus, formal training may increase confidence in the ability to use technology and consequently improve overall success.

Care staff shared the same experiences when it came to supporting an Autistic individual to use technology:

Gary: I think the problem that we seem to have is that putting a piece of technology in can be very complicated…if I go back to Harry – that’s certainly what he felt with his equipment. Staff found it really hard because it was all set up, it was all ready to go, staff didn’t know how to use it and he was getting frustrated with it

Gary: Very little training…when it was speaking it wasn’t very loud and he had this frustration. Obviously there are other things out there that we’d be able to use it for but I think there’s a lack of knowledge, that, I’m sure there’s hundreds of stuff out there that we’d be able to use but without the knowledge of what’s out there we’re very much restricted, like if someone says have you tried that? No we haven’t!

You’re in a position where if anything goes wrong you go round in a big huge circle to get someone out to help you & actually there’s no, yeah there’s no training, there’s no quick guide on how to fix anything

Care staff went on to discuss how funding issues create an additional barrier to training:

Jean: that’s what we hit all the time in social care..it’s about funding everything.

Gary: The same as every other organisation at this moment in time, the local authority are looking at the cheapest way possible of providing that support..so what you might find or what we’ve experienced in the past is that we get something…that’s a kind of step in the right direction but they don’t go the full way because they don’t want to spend the money they just need to go through the process of trying things before they put in the technology to make it work

These concerns regarding lack of funding reflect the current economic climate as there are increasing financial strains both on the National Health Service (NHS) and third sector organisations who provide care for Autistic individuals (Pellicano et al., 2014). Despite support staff wanting to support individuals to use technology they are limited by a lack of funding and training. So although public money is spent on the development of technology there is limited funds on the support, training and implementation methods that should coincide this advancement (Ravet, 2015).

#### Sub-theme: Defining Individual Goals and Needs

**‘***How***’** technology is implemented can often determine whether or not it is successful and may be key in overcoming some of the barriers discussed above. Consistency in how staff implement technologies within an individual’s life was highlighted as a key contributing factor to successful technology use. There was consensus that a person centered approach to implementing technological solutions and more robust strategies to ‘get staff teams on board’ would improve overall success (e.g. bespoke training, regular team meetings that include the person being supported, and standardized follow-up evaluations).

Ann: N they’re there 24 h if your gonna make something work you’ve got to involve the team

Paula: There’s no budgets anymore for the people we support n they are looking at the different options of technology…reducing hours n stuff n we need support staff to come on board with it to give them more positives out it

Ann: aye..so its staff getting on board with it I think..can be a hurdle sometimes aswell

Participants also shared experiences of supporting Autistic individuals, who would normally receive 24 h care, however, have had their support hours cut due to a lack of funding and are therefore on their own during the night or for a period of the day. Discussions evolved to consider how technological solutions may aid safety and well-being in these circumstances if they are implemented in the correct ways.

Participants discussed how many third sector care organisations are turning to technological solutions as a means of ‘cutting costs’ and that in order for them to be successful, staff teams need to be on board and consistent in the delivery of such technology. This means implementing technology in a person centered way, for example someone who isn’t keen on change or trying new things should have technological interventions implemented slowly and over a period of time, allowing for formal training for both the Autistic person, families and staff to be put in place.

One Autistic participant further stressed the need for individualized support strategies:

Alex: So often it’s a case of, you know, you get given a lot of help which makes you feel babied or incompetent or you get the, just suck it up and get on with it.

These experiences emphasize the importance of defining individual goals, needs and preferences as part of the implementation process, highlighting the importance of co-production methods.

## Limitations

A limitation of the current study was that participants knowledge of existing technologies was not measured. Participants with more or less knowledge than others in the wider Autistic community, may have different experiences or expectations of emotion regulation technology. Also, the focus groups were carried out across different contexts i.e. home, community care and education. This may have caused differences in the amount of information (especially critical) that was provided to the researcher. In addition, to achieve a multi-disciplinary perspective, smaller groups of each discipline were recruited, therefore results may not extend to a wider population. Although the study did not recruit a large sample, fewer participants resulted a more in depth analysis. Future research may wish to examine one group only to avoid too many variations of opinion and perspective. Despite these limitations however, the current study shows that there is much consensus between the groups on the benefits and issues surrounding technological supports aiming to regulate emotions for autistic people.

## Conclusion and Implications

First, this study aimed to provide meaningful insight into experiences and expectations of technology that aimed to regulate emotions for Autistic people. Results show that the success of technology in helping to support emotion regulation is impacted by how adaptable, affordable, and easy to use it is. For example, participants felt that most technology is not ‘easy to use’. Care and educational staff discussed how individuals are ‘often reluctant to use technology’ due to a lack of formal training. Parents of children with Autism expressed how they would only try new technology to support emotion regulation if it wasn’t too complicated, low-cost, and specific to their child’s needs. This is further confounded by socio-economic factors, resulting in limited access to technology or knowledge about availability. Moving forward, the implementation of technology could be improved so that end users have confidence in their ability to utilize the technology and feel that it is suited to their ability/development: this was highlighted extensively across all focus groups, and across the variety of contexts (i.e. home, education, and care settings).

The second aim was to explore participant’s views on the advantages and disadvantages of real time emotion regulation technology based on physiological signals. Differences in opinions between participant groups were highlighted. For example, some Autistic participants felt there is a lack of technology aimed towards Autistic individuals with higher IQ, however did suggest that real time emotion regulation technology may be more beneficial for Autistic individuals with more complex needs e.g. additional learning needs or those at increased vulnerability (i.e. self-harm, aggressive outbursts etc.). This emphasizes the importance of personalized technological support that is flexible and can adapt to the varying abilities and needs of Autistic individuals. Data also emphasized the need for emotion regulation technology that does not try to replace Autistic traits with ideal states. Future research in this area must place Autistic people at the heart of technology development if it is to be successful.

Thirdly, the paper aimed to provide a basis for improving individualised and interactive emotion regulation technology for Autistic people. Based on the findings, a number of recommendations are proposed: (1) With regards to technology design, usability is likely to increase through participatory methods/by adopting co-design approach at every stage of both the research process and technology development. There was consensus expressed to provide simple, easy to use and adaptive technology to meet individual needs. (2) Implementation strategies that take the individuals’ life goals into consideration in a more holistic and person centered way is needed, this would include bespoke training procedures for guidance for families, staff, and individuals. (3) An important and innovative direction for future research in this area would be to investigate the use of physiological signals for the development of interactive sensor technology able to support self emotion regulation strategies. Whilst this area of research has many positive implications, ethical challenges must be addressed and an emphasis must be placed on co-participatory methods to develop technological supports that can adapt to a wide variety of abilities, promote independence, empower, and improve socio-emotional well-being for Autistic people.
